# Sex-specific differences in hepatic steatosis in obese spontaneously hypertensive (SHROB) rats

**DOI:** 10.1186/s13293-018-0202-x

**Published:** 2018-09-10

**Authors:** Qingming Dong, Michael S. Kuefner, Xiong Deng, Dave Bridges, Edwards A. Park, Marshall B. Elam, Rajendra Raghow

**Affiliations:** 1 Department of Veterans Affairs Medical Center, 1030 Jefferson Avenue, Memphis, TN 38104 USA; 2 Department of Pharmacology, College of Medicine, The University of Tennessee Health Science Center, 874 Union Avenue, Memphis, TN 38163 USA; 3 Department of Nutritional Sciences, University of Michigan School of Public Health, Ann Arbor, MI 48109 USA; 4 Department of Pharmacology, College of Medicine, University of Tennessee Health Science Center, 874 Union Avenue, Memphis, TN USA

## Abstract

**Background:**

Patients with metabolic syndrome, who are characterized by co-existence of insulin resistance, hypertension, hyperlipidemia, and obesity, are also prone to develop non-alcoholic fatty liver disease (NAFLD). Although the prevalence and severity of NAFLD is significantly greater in men than women, the mechanisms by which gender modulates the pathogenesis of hepatic steatosis are poorly defined. The obese spontaneously hypertensive (SHROB) rats represent an attractive model of metabolic syndrome without overt type 2 diabetes. Although pathological manifestation caused by the absence of a functional leptin receptor has been extensively studied in SHROB rats, it is unknown whether these animals elicited sex-specific differences in the development of hepatic steatosis.

**Methods:**

We compared hepatic pathology in male and female SHROB rats. Additionally, we examined key biochemical and molecular parameters of signaling pathways linked with hyperinsulinemia and hyperlipidemia. Finally, using methods of quantitative polymerase chain reaction (qPCR) and western blot analysis, we quantified expression of 45 genes related to lipid biosynthesis and metabolism in the livers of male and female SHROB rats.

**Results:**

We show that all SHROB rats developed hepatic steatosis that was accompanied by enhanced expression of SREBP1, SREBP2, ACC1, and FASN proteins. The livers of male rats also elicited higher induction of *Pparg*, *Ppara*, *Slc2a4*, *Atox1*, *Skp1*, *Angptl3*, and *Pnpla3* mRNAs. In contrast, the livers of female SHROB rats elicited constitutively higher levels of phosphorylated JNK and AMPK and enhanced expression of *Cd36*.

**Conclusion:**

Based on these data, we conclude that the severity of hepatic steatosis in male and female SHROB rats was mainly driven by increased de novo lipogenesis. Moreover, male and female SHROB rats also elicited differential severity of hepatic steatosis that was coupled with sex-specific differences in fatty acid transport and esterification.

**Electronic supplementary material:**

The online version of this article (10.1186/s13293-018-0202-x) contains supplementary material, which is available to authorized users.

## Background

Metabolic syndrome (MetS), a sequela characterized by the co-occurrence of insulin resistance, hypertension, hyperlipidemia, and obesity [1], is emerging as a major public health challenge, affecting nearly one third of adults in the USA of America [2]. Epidemiological studies have clearly demonstrated that although both genetic and environmental factors underlie the development of MetS, its primary drivers are overconsumption of calorie-dense foods and sedentary lifestyle. Clinical correlates of MetS and its molecular underpinnings have been extensively investigated in rodent models such as *ob/ob* mice [3], *db/db* mice [4], and *fa/fa* rats [5]. Although it is well known that rodent models of MetS mimic the human syndrome to varying degrees [6], studies in rodents have yielded critical evidence that mechanistically links hallmarks of MetS (visceral adiposity, hyperglycemia, and hypertension), with mechanisms of nutrient partitioning and energy homeostasis. Experimental animals have also played a crucial role in unraveling the genetic, neuronal, and humeral mechanisms involved in the control of food intake and energy metabolism and how their defective regulation leads to MetS [7–10].

The obese spontaneously hypertensive (SHROB) rats, also known as Koletsky rats, are thought to manifest the essential traits that characterize the MetS [11]. The SHROB rats were shown to be unique since they not only elicited genetic hypertension similar to their lean, spontaneously hypertensive (SHR) parents but also elicited congenital obesity on regular chow diet. The genome of SHROB rat harbors a nonsense mutation in codon 763 of the gene encoding the leptin receptor [12]. SHROB rats express normal amounts of mutated leptin receptor messenger RNA that fails to be translated into functional leptin receptors [13–15]. In the face of harboring two copies of the defective leptin receptor gene, SHROB rats were also shown to hyper-secrete leptin in their bloodstream. Thus, besides being hypertensive, SHROB rats elicited monogenetic obesity that was associated with hyperlipidemia, hyperinsulinemia, and striking proteinuria and renal disease [11]. It is also noteworthy however, that although SHROB rats were glucose intolerant compared with their heterozygous or WT littermates, their fasting glucose levels were normal even on a high-sucrose diet. Strikingly, both male and female SHROB rats were infertile and had a shorter lifespan, surviving only for 10–11 months while their WT littermates had an average lifespan of 2–3 years [16, 17].

The prevalence and severity of non-alcoholic fatty liver disease (NAFLD) in many populations around the globe is higher in men than women [18–20]. Although some gender-specific differences in the metabolism of dietary glucose and fatty acids in humans have been reported [21], how they differentially modulate the pathogenesis of hepatic steatosis and NAFLD is far from clear [22]. SHROB rats are known to develop hepatomegaly and fatty liver similar to many patients with MetS. Several investigators have peered into the hormonal and biochemical underpinnings of adiposity and MetS in SHROB rats [23–25]. For instance, Ernsberger et al. observed widespread deposition of fat in the SHROB rats but noted that their retroperitoneal and subscapular adipose depots were particularly prominent [26]. These authors also reported that, in contrast to their SHR counterparts, the adiposity of SHROB rats was apparently affected by their sex; they showed that while SHROB males accumulated nearly 12-fold higher amount of epididymal fat, the myometrial fat depots in female SHROB rats were elevated by 19-fold. Based on the rates of incorporation of labeled glucose into lipids in various tissues (e.g., muscle, adipose tissue, and liver), Ernsberger et al. surmised that obesity of SHROB rats was mainly driven by de novo lipid synthesis [26]. Although these observations hinted at the possibility of sexual dimorphism underlying the development of MetS, its putative sex-specific consequences on the metabolism of lipids in the liver of SHROB rats were not studied in depth. To address this question, we examined the signaling pathways canonically linked with hyperinsulinemia in the livers of male and female SHROB rats. Additionally, we compared the expression of a set of 45 candidate genes that are known to be involved in sex-specific regulation of the biosynthesis and metabolism of lipids. Our findings corroborate and extend previous studies to indicate that hepatic steatosis and its molecular mechanisms are indeed differentially regulated in male and female SHROB rats.

## Methods

### Reagents

Anti-IRS-1, anti-pIRS-1 (S307), anti-AKT (T308), anti-AKT, anti-p-mTOR (S2448), anti-mTOR, anti-p-p70S6K (T389), anti-p70S6K, anti-p-S6 (S235/S236), anti-S6, anti-p-p38MAPK (T180/Y182), anti-p38MAPK, anti-p-ERK1/2 (T202/Y204), anti-ERK1/2, anti-p-SAPK/JNK (T183/Y185), anti-SAPK/JNK, anti-p-AMPK (T172), anti-AMPK, anti-ACC1, and anti-FASN monospecific antibodies were purchased from Cell Signaling (Danvers, MA). Insulin, Oil Red O, and anti-actin antibodies were obtained from Sigma-Aldrich (St. Louis, MO). Anti-SREBP1 antibody was bought from Becton-Dickinson (Franklin Lakes, NJ). Anti-SREBP2 and anti-ChREBP antibodies were bought from R&D Systems (Minneapolis, MN). Combined protease and phosphatase inhibitor cocktails (Halt™) and Tissue-Tek™ optimum cutting temperature (OCT) compound were bought from Thermo Fisher Scientific (Hampton, NH). Forward and reverse oligonucleotide primers (Additional file 1: Table S1) to carry out quantitative PCR analysis were designed according to a published program (OligoArchitectTM online, Sigma-Aldrich, St. Louis, MO). DNA primers were synthesized by Integrated DNA Technologies (Coralville, IA).

### Animal husbandry, genotyping, and laboratory studies

All animal procedures were approved by the Institutional Animal Care and Use Committee (IACUC) of University of Tennessee Health Science Center (UTHSC), Memphis. Adult male and female heterozygous SHROB breeding pairs were obtained from a closed colony that has been continuously maintained since 1973 [26]. Since homozygous rats are sterile, the SHROB strain was propagated by mating age-matched heterozygous males and females. Rats were housed in a room with a constant light and dark cycle of 12-h each, at 20–23 °C. Animals had full access to Teklad LM-485 mouse/rat chow (containing 17, 25, and 58% calories from fat, protein, and carbohydrate, respectively), bought from Research Diets Inc. (New Brunswick, NJ). Most experimental measurements, signal transduction, and gene expression analyses (western blot and qPCR) were carried out on 10-week-old homozygous male and female SHROB rats.

Tail clip DNA samples, extracted using DNase Blood & Tissue Kit (Qiagen, Hilden, Germany), were subject to PCR with tetra-pair ARMS (amplification-refractory mutation system) primers (Additional file 1: Table S1) that were designed as described in detail (http://primer1.soton.ac.uk/primer1.html). The PCR was done using Thermo Scientific DreamTaq PCR Master Mix (2×) in a SimpliAmp Thermal Cycler (Applied Biosystems by Life Technologies, Foster City, CA). The PCR conditions were 94°C for 3 min, 35 cycles of 94°C 1 min, 51.1°C 1 min, and 72°C 1 min, followed by extension at 72°C for 10 min. DNA fragments containing codon 763 of the leptin receptor gene were amplified with two outer primers (Additional file 1: Figure S1) and purified by ExoSAP-IT™ PCR Product Cleanup Reagent from Thermo Fisher Scientific (Waltham, MA). Amplified genomic DNA fragments were sequenced in the Molecular Resource Center of the UTHSC, using ABI Model 3130XL Genetic Analyzers (Foster City, CA) with four-color fluorescence-based sequencing.

Body weight was measured weekly between weeks 3 and 10. Total fat mass and fat-free mass of WT and homozygous SHROB rats were measured at week 10, using a magnetic resonance imaging (MRI) machine, EchoMRI-1100 (EchoMRI LLC, Houston, TX). Blood was collected from 10-week-old rats via cardiac puncture, and serum was obtained from clotted blood. Concentrations of alanine aminotransferase (ALT), amylase, aspartate aminotransferase (AST), blood urea nitrogen (BUN), creatinine, cholesterol, glucose, and triglyceride in the sera were determined by IDEXX BioResearch Laboratories (Columbia, MO).

### Western blot analysis

Protein extracts from homogenized livers were size-fractionated by SDS-PAGE and transferred onto nitrocellulose membranes that were probed with monospecific, primary, and secondary antibodies as described previously in detail [27]. Western blots were quantified using Quantity One software from Bio-Rad (Hercules, CA).

### Quantification of gene expression by qPCR

Three replicate liver samples, representing WT and SHROB male and female rats, were used to extract total RNA using Trizol reagent (Invitrogen, Carlsbad, CA). RNA was converted into cDNA and subject to quantitative PCR (qPCR) with gene-specific primers (Additional file 1: Table S1) and GoTaq qPCR Master Mix (Promega, Madison, WI). PCR amplification was carried out using a LightCycler 480 Real-Time PCR system (Roche, Basel, Switzerland). The specificity of the qPCR amplification was verified by melting curve analysis. Data were analyzed using the ΔΔCT threshold cycle method. Target mRNAs were normalized against *Rn18s* ribosomal RNA and fold changes in experimental samples relative to those in control samples were calculated [28].

### Histology

Tissue samples fixed in 4% paraformaldehyde/PBS for 24 h and embedded in paraffin were cut into 5 μm sections. De-paraffinized liver sections were stained with hematoxylin and eosin (H&E). Blocks of fresh liver tissues were embedded in frozen OCT compound and cryo-sectioned into 14-μm-thick slices; tissue sections were stained with 0.3% Oil Red O for 5 min at room temperature to visualize intracellular accumulation of lipid droplets [29]. The Oil Red O staining of tissue sections was quantified using the ImageJ software (NIH, Bethesda, MD).

### Extraction and measurement of triglyceride content in liver tissue

Measurements of hepatic TG content were done exactly as outlined in detail previously [30]. Fragments of frozen liver were taken up in homogenization buffer (50 mM Tris, pH 8, 5 mM EDTA, 30 mM mannitol, and cocktail of protease inhibitors) and lysed using a TissueLyser II (Qiagen). Total lipids were extracted with 250 mM KOH and a mixture of chloroform and methanol (2:1). Liver tissue extracts were centrifuged at 13,000 × *g* for 10 min. As described by Lu et al. [30], the bottom layer was dried and taken up in a mixture of 3 mL butanol, 1.66 mL Triton X-114, and 0.33 mL methanol, and TGs were quantified using the Sigma Triglyceride Assay Kit (Cat TR0100, Sigma-Aldrich).

### Statistical analysis

Means ± SEM were derived from 3 to 5 experiments (as indicated in the figure legends). A two-way analysis of variance (ANOVA) was performed to assess statistically significant differences between groups, using GraphPad Prism 6.0 software (La Jolla, CA). *p* values of < 0.05 were considered to be statistically significant. Putative interactions between genotype and gender were similarly examined by two-way ANOVA measurements.

## Results

### Genotyping of SHROB rats

SHROB rats were genotyped using a strategy based on PCR amplification of genomic DNA followed by its sequencing (Additional file 1: Figure S1a and b). We designed four PCR primers to amplify a region of the leptin receptor genomic DNA that included T > A mutation that leads to premature termination of translation in the leptin receptor mRNA [13]. Amplification of the genomic DNA by PCR using flanking primers should yield a 248-bp DNA fragment regardless of the genotype whereas two internal primers should yield 184-bp and 118-bp fragments representing WT or SHROB alleles, respectively (Additional file 1: Figure S1a). As expected, genomic DNA of heterozygous rats produced three fragments, 248-bp, 184-bp, and 118-bp in length; in contrast, PCR amplification of homozygous SHROB genomic DNA produced 248-bp and 118-bp bands (data not shown). Sequencing of DNA around codon 763 of the leptin receptor gene unequivocally identified the genotypes of the WT, heterozygous, and homozygous rats (Additional file 1: Figure S1b).

### Gender-specific differences in body mass and liver size in SHROB rats

**Fig. 1:**
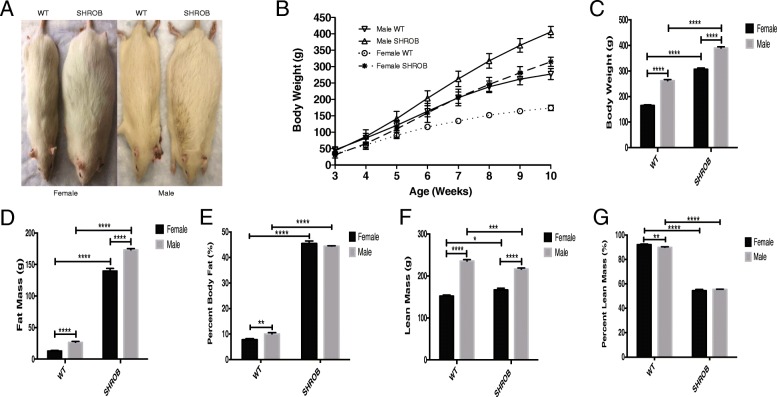
Gender-specific comparison of body composition in WT and SHROB rats. **a** Representative gross morphology of 10-week-old female and male WT and SHROB rats. **b** Body weight curves of various groups between week 3 and week 10. **c** Body weight at week 10. **d** Fat mass at week 10. **e** Percent body fat at week 10. **f** Lean mass at week 10. **g** Percent lean mass at week 10. Data are presented as mean ± SEM. *n* = 5 to 12 for each group. Statistical significance: *p < 0.05, **p < 0.01, ***p < 0.001, and ****p < 0.0001

We assessed rates of growth and body composition of female and male SHROB rats fed with a regular chow diet, between the ages of 3 and 10 weeks. As reported originally [11], the obese phenotype is recessive, and the gross morphology of heterozygous (Fa^k^/fa^k^) rats did not differ significantly from WT. Therefore, in our study, all comparisons were made either between WT and homozygous (fa^k^/fa^k^) SHROB rats or between SHROB (fa^k^/fa^k^) males and females. A representative WT and SHROB female and male rats at 10-week of age are shown in Fig. 1a. Body weights of 3-week-old rats, irrespective of their genotypes, were very similar. Additionally, although all animals steadily grew bigger over a period of 7 weeks, the growth curve of the SHROB male rats was the steepest among the four groups of animals; in contrast, WT females elicited the lowest rates of body mass gain (Fig. 1b). At week 10, males were visibly bigger than females (Fig. 1a–c).

**Fig. 2:**
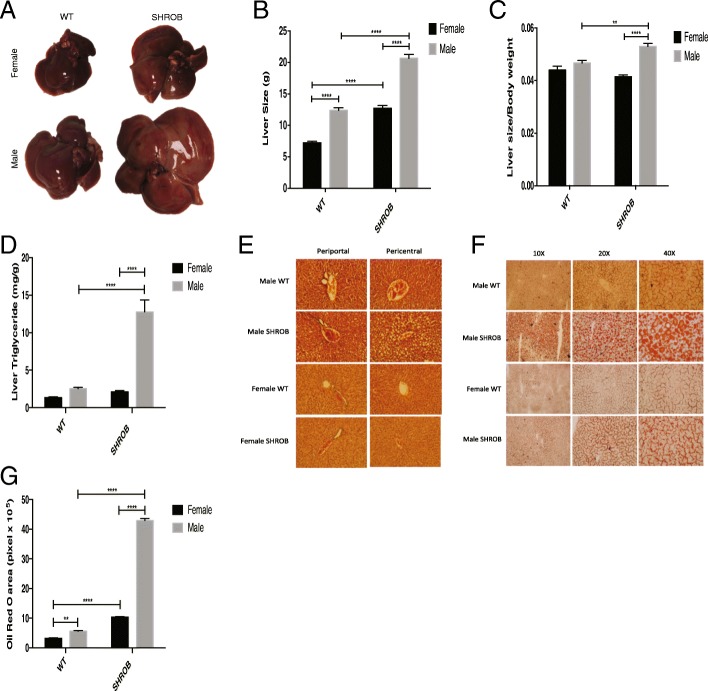
Morphological and histological comparisons of the livers from WT and SHROB rats. **a** Representative gross morphology of the livers from male and female WT and SHROB rats at week 10 of age. Liver weight (**b**) and liver weight normalized against total body weight (**c**) in WT and SHROB rats at week 10 is shown. Each group had between 5 and 12 rats. **d**, **e** Representative microphotographs of female and male WT and SHROB rat livers stained with H&E (**d**) and Oil Red O (**e**). **f** Quantification Oil Red O staining. **g** Results are presented as the means ± SEM of three separate experiments; × 20 magnification is used for quantification. *n* = 3 for each group. Statistical significance: **p < 0.01, ****p < 0.0001

Next, we measured relative changes in the total body fat and lean masses of WT and SHROB rats at week 10. Compared with WT rats, the fat mass of female and male SHROB rats was increased by 10.8- and 6.5-fold, respectively (Fig. 1d). Although, total fat mass of male SHROB rats was higher versus homozygous females (Fig. 1d), when expressed as percentage of body weight, fat mass increased to comparable levels in male and females (Fig. 1e). Moreover, although lean mass for both male WT and SHROB rats were higher than corresponding females, 10-week-old male SHROB rats lost significant lean mass compared to WT. In contrast, 10-week-old SHROB females gained lean mass compared with their WT female littermates (Fig. 1f). We should also note that at week 10, percent lean mass in both male and female SHROB rats was significantly decreased compared with WT rats, respectively (Fig. 1g). The results of two-way ANOVA (Figs. 2d–g) indicated that both genotype and gender contributed to age-dependent changes in fat mass, percent body fat, lean mass, and percent lean mass of SHROB rats fed a regular chow diet.

Visible gender-specific differences in the sizes of bodies of SHROB rats corresponded with the sizes of their livers (Fig. 2a, b). We also noted that although livers of both female and male SHROB rats grew bigger at week 10, the livers of male rats were larger and paler in appearance (Fig. 2a). The apparent disparity in sizes of livers between male and female SHROB rats remained highly significant even after the normalization of their liver weights against body weights (Fig. 2c). These data revealed that, as expected, the livers of male SHROB rats were considerably larger than their male WT littermates. Even more significantly, the livers of male SHROB rats were even bigger and paler than those of SHROB females (Fig. 2a–c). To assess the underlying biochemical basis of differential gross morphology (more lipid droplets), we measured hepatic TG content in these animals. As shown in Fig. 2d, the livers of male SHROB rats had ~ 6-fold greater amount of TG compared with females (Fig. 2d).

To assess if gender-specific differences in gross morphology of male SHROB rats and their livers were related to putative changes in hepatic tissue architecture, we sectioned the livers from male and female SHROB rats and stained these with H&E and Oil Red O. As shown in Fig. 2e, H&E-stained sections of livers from male SHROB rats appeared highly vacuolated (denoting areas of lipid droplets accumulation, as judged by Oil Red O staining). Lipid-laden hepatocytes were more prevalent in the pericentral areas of the liver as opposed to periportal areas (Fig. 2e). Thus, Oil Red O staining revealed that although some hepatic steatosis was evident in all SHROB rats (unlike WT animals) regardless of their gender (Fig. 2f), SHROB males elicited more severe steatosis as judged by accumulation of lipid droplets (Fig. 2f). Quantification of lipid droplets confirmed the visual impression to reveal disproportionately greater accumulation of fat in the livers of male SHROB rats (Fig. 2g). We should note however, that in spite of the differences in the hepatic histology of male and female SHROB rats, H&E staining did not reveal signs of overt fibrosis (Fig. 2e; data not shown). However, these data led us to tentatively conclude that both genotype and gender contributed to the severity of hepatic steatosis in SHROB rats.

### Biochemical analysis of serum in female and male SHROB rats

**Fig. 3:**
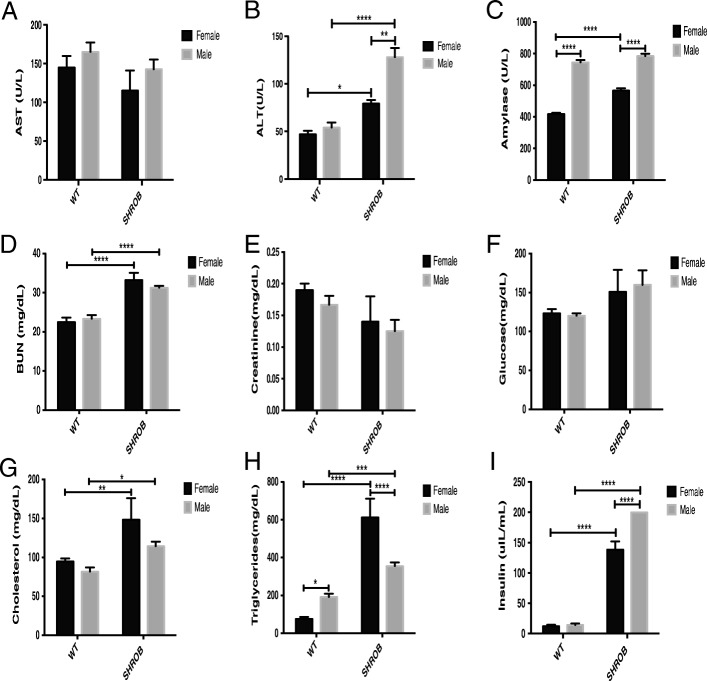
Serum chemistries for 10-week-old female and male WT and SHROB rats. Data are mean ± SEM. *n* = 5 to 12 for each group. **a** AST, aspartate aminotransferase. **b** ALT, alanine aminotransferase. **c** Amylase. **d** BUN, blood urea nitrogen. **e** Creatinine. **f** Glucose. **g** Cholesterol. **h** Triglyceride. **i** Insulin. Statistical significance: *p < 0.05, **p < 0.01, ***p < 0.001, and ****p < 0.0001

We analyzed the serum of male and female SHROB rats for markers of hepatic inflammation, pancreatic and kidney functions, and circulating lipids. As shown in Fig. 3, the levels of AST, creatinine, and glucose were very similar in all groups of rats. Compared to WT rats, serum ALT levels were 1.7-fold and 2.4-fold higher in female and male SHROB rats, respectively (Fig. 3b).

The amylase index for both male WT and male SHROB rats was higher than the respective females (Fig. 3c). Compared with WT rats, the levels of BUN were greater in both male (~ 34%) and female (~ 48%) SHROB rats (Fig. 3d). In 10-week-old rats, there were small but insignificant changes in the levels of creatinine and glucose; these animals have been shown to develop proteinuria and renal disease in later life [11]. Both male and female SHROB rats had hypercholesterolemia compared with WT rats (Fig. 3g). The levels of TGs in circulation in female SHROB rats were ~ 8-fold higher compared to WT females. We should note however, that serum TGs were only 1.85-fold higher in the SHROB males (Fig. 3h). Compared with their WT littermates, hyperinsulinemia was evident in both male and female SHROB rats, with circulating levels of insulin being 14.1-fold and 11.5-fold higher, respectively (Fig. 3i).

### Insulin pathway is differently influenced for female and male SHROB rats

**Fig. 4:**
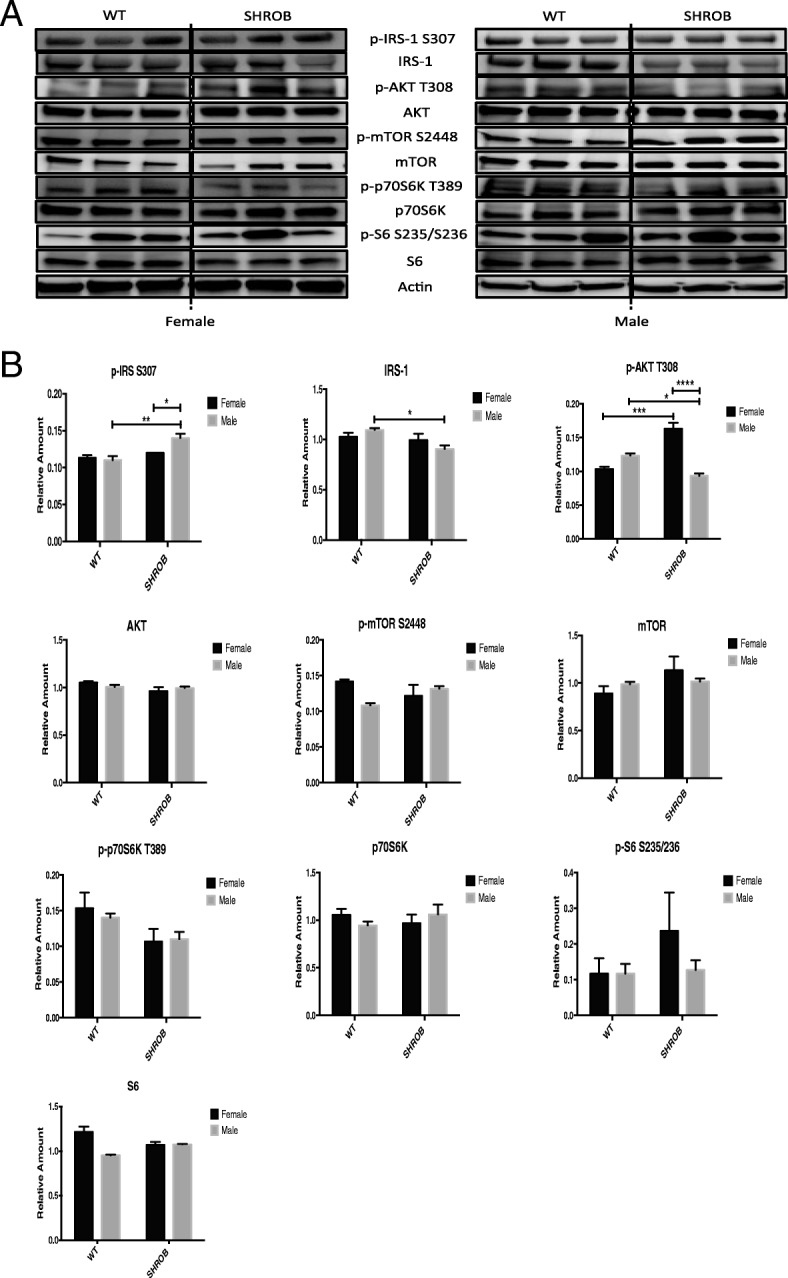
Analysis of canonical insulin and mTOR signaling pathways in the livers of female and male WT and SHROB rats. **a** Western blots of total and phosphorylated proteins from three rat livers for each group are shown. **b** Densitometry of polypeptide band (panel **a**) was used to calculate the amount of target proteins and was normalized against actin. Statistical significance: *p < 0.05, **p < 0.01, ***p < 0.001, and ****p < 0.0001

Since both hyperinsulinemia and hepatic steatosis were more pronounced in male SHROB rats, we investigated if insulin signaling pathways were differentially impaired in males and females. It has been documented previously [26] that the skeletal muscle and livers of SHROB rats had lower levels of total and phosphorylated insulin receptor substrate 1 (IRS-1). Consistent with earlier reports, we noted that levels of IRS-1 in the livers of SHROB rats were significantly lower (Fig. 4). IRS-1, a key target of insulin receptor kinase, contains more than 30 potential serine/threonine phosphorylation sites; while phosphorylation of some sites (e.g., Y895, Y1222) is associated with activation of IRS-1, phosphorylation of others (e.g., S307) disrupts IRS-1/insulin receptor interaction [31–33]. As shown in Fig. 4, hepatic extracts of male SHROB rats had higher steady-state levels of IRS phosphorylated at S307, reflecting an impairment of insulin signaling.

IRS-1 phosphorylates and activates PI3 kinase that generates PIP_3_ and activates the 57-kDa Ser/Thr kinase AKT (also known as protein kinase B, PKB) via phosphorylation at T308. As judged by pAKT (T308) levels in the hepatic extracts of SHROB females (Fig. 4), they elicited greater insulin sensitivity compared with their male counterparts. We should note however, that despite gender-specific differences seen in the proximal steps of insulin signaling pathway, the downstream activation of the mTOR (mechanistic target of rapamycin) signals was apparently elicited in a gender-neutral fashion. Thus, we did not observe significant differences in the constitutive levels of total or phosphorylated mTOR, p70S6K, and S6K in the liver extracts obtained from male and female SHROB rats (Fig. 4).

**Fig. 5:**
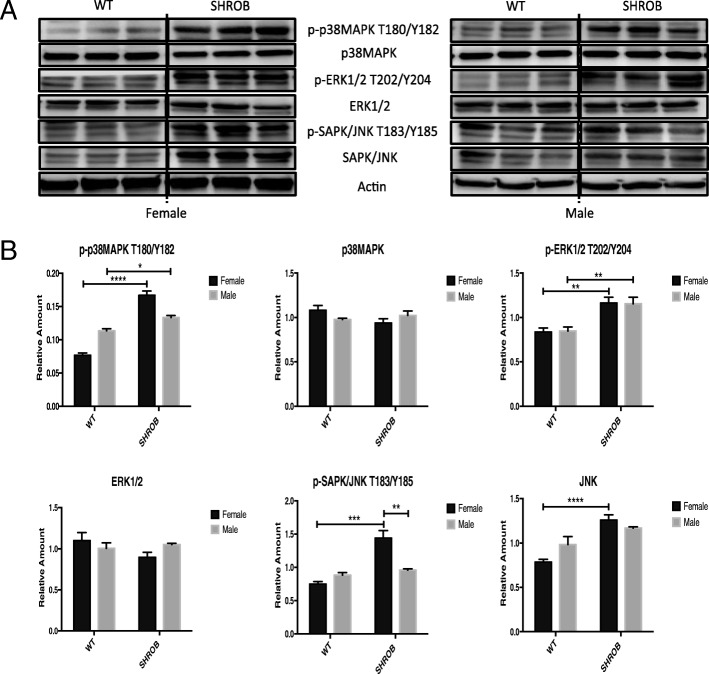
Western blots and densitometry for MAPK pathway in female and male WT and SHROB rat livers. **a** Western blots. **b** Densitometry data from panel **a** showing relative amount of total proteins, p-ERK1/2T202/Y204 and p-SAPK/JNK T183/Y185 after their normalization against actin. The relative amount of p-p38MAPK T180/Y182 was normalized against p38MAPK level and actin. Statistical significance: *p < 0.05, **p < 0.01, ***p < 0.001, and ****p < 0.0001

Crosstalk between PI3K-AKT/PKB and MAPK signaling is known to be significantly altered during hyperinsulinemia [34]. Therefore, we assessed putative changes in the activation of MAPK pathways and their downstream consequences for hepatic gene expression in female and male SHROB rats (Fig. 5). We discovered that SHROB rats of both genders had higher levels of p-p38MAPK (phosphorylated at T180/Y182) and p-ERK1/2 (phosphorylated at T202/Y204) compared to their respective WT counterparts. In contrast, hepatic extracts of female SHROB rats had higher constitutive levels of activated SAPK/JNK, phosphorylated at T183/Y185 (Fig. 5).

**Fig. 6:**
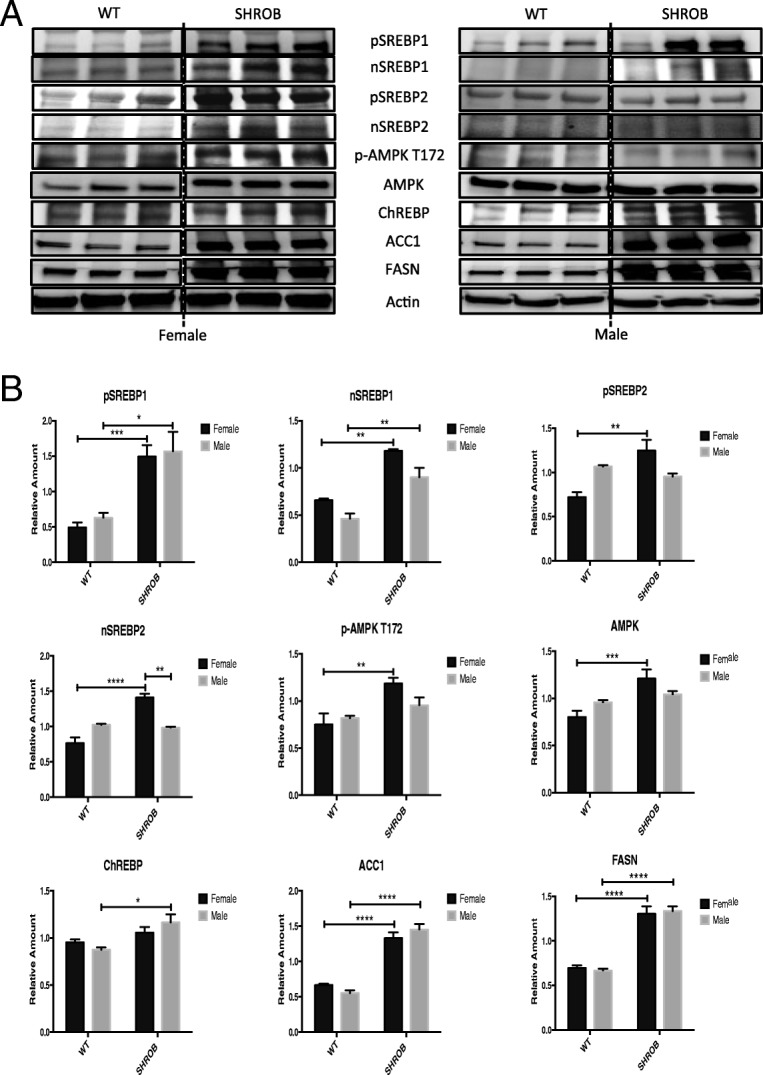
Western blot analysis and quantification of lipogenesis proteins in the livers of WT and SHROB rats. **a** Western blots for female/male WT and SHROB rat livers sacrificed at week 10, *n* = 3 for each group. **b** Densitometry data from (panel **a**). The relative amount of total proteins and p-AMPK T172 is based on target gene expression level normalized by actin. Statistical significance: *p < 0.05, **p < 0.01, ***p < 0.001, and ****p < 0.0001

Since the canonical PI3K-AKT/PKB and MAPK signaling pathways culminate in the nucleus by inducing a program of gene expression relegated to de novo lipid synthesis and gluconeogenesis, we quantified expression of a subset of metabolic regulators by western blot analysis, followed by densitometry (Fig. 6). These experiments revealed that the steady-state levels of proteins involved in de novo lipid biosynthesis (pSREBP1, nSREBP1, ACC1, and FASN) were expressed more abundantly in the livers of SHROB rats. We also noted however, that female SHROB rat livers elicited greater expression of pSREBP2, nSREBP2, p-AMPK, and AMPK (Fig. 6). In contrast, the expression of ChREBP (carbohydrate response element-binding protein), a key transcription factor involved in de novo lipid synthesis [35, 36] was more abundantly expressed in the livers of SHROB males. Interestingly, the steady-state levels of total and phosphorylated AMP-activated protein kinase (AMPK) were higher in the livers of SHROB females (Fig. 6).

### Quantification of gene expression by qPCR in WT and SHROB rats

**Fig. 7:**
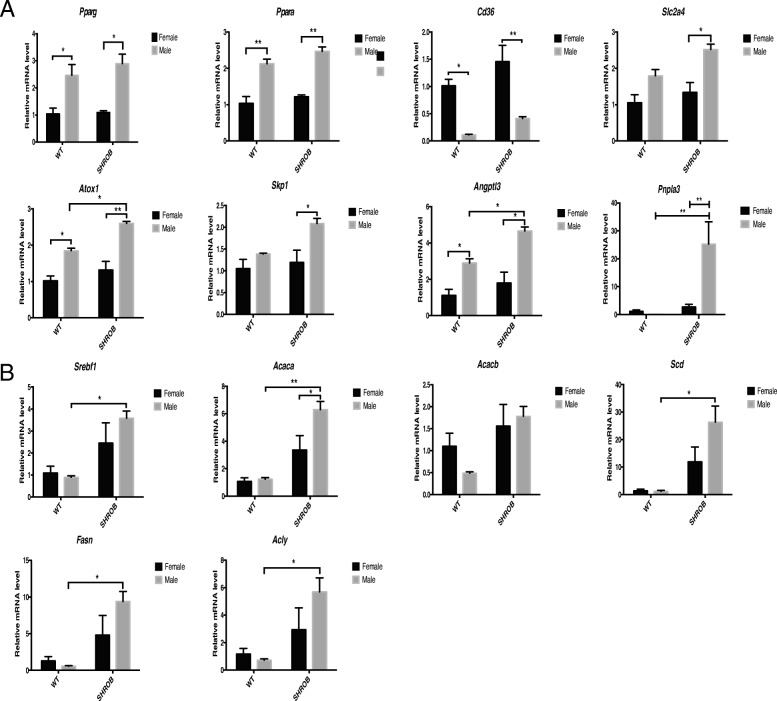
Quantification of gender-specific differences in hepatic gene expression by qPCR in SHROB rats. The relative mRNA levels were normalized against Rn18s RNA and compared with WT female set as 1. *N* = 3 for each group. Statistical significance: *p < 0.05, **p < 0.01. **a** Genes related to gender differences including peroxisome proliferator-activated receptor gamma (*Pparg*), peroxisome proliferator-activated receptor alpha (*Ppara*), cluster of differentiation 36 (*Cd36*), solute carrier family 2 member 4 (*Slc2a4*), antioxidant protein 1 (*Atox1*), S-phase kinase-associated protein 1 (*Skp1*), angiopoietin-like 3 (*Angptl3*), and papatin-like phospholipase domain-containing protein 3 (*Pnpla3*). **b** Genes related to lipogenesis including sterol regulatory element-binding transcription factor 1 (*Srebf1*), acetyl-CoA carboxylase alpha (*Acaca*), acetyl-CoA carboxylase beta (*Acacb*), stearoyl-CoA desaturase (*Scd*), fatty acid synthesis (*Fasn*), and ATP citrate lyase (*Acly*)

To further investigate the putative molecular basis of sexual dimorphism in hepatic lipid metabolism in SHROB rats, we assessed the mRNA levels of a panel of 45 genes shown previously to be differentially regulated in males and females [37]. As shown in Fig. 7, the expression of *Pparg*, *Ppara*, *Slc2a4*, *Atox1*, *Skp1*, and *Angptl3* genes was moderately increased in the female SHROB rats; however, these animals elicited much greater expression of *Cd36* compared with SHROB males. Moreover, although steady-state levels of mRNAs encoded by *Srebf1*, *Acaca*, *Scd*, *Fasn*, and *Acly* were increased in both male and females, and their expression was significantly greater in males; a ~ 500-fold induction of *Pnpla3* expression in SHROB males was particularly notable (Fig. 7).

The four cardinal features of MetS in humans, insulin resistance, visceral adiposity, severe dyslipidemia, and endothelial dysfunction are often associated with systemic inflammation [6]. Therefore, we investigated if expression of canonical pro-inflammatory genes (e.g., *Tnf*, *IL-1b*, *Il-6*, and *Ccl2)* in the livers of male and female SHROB rats were differentially affected. As judged by the results of qPCR, none of the target genes showed gender-associated differential expression of the livers of WT or SHROB rats (data not shown).

## Discussion

Non-alcoholic fatty liver disease (NAFLD) is frequently associated with MetS [38]. The initial stage of NAFLD is hepatic steatosis, which may progress to non-alcoholic steatohepatitis [38] and eventually hepatocellular carcinoma [39]. Both epidemiological and animal studies indicate that the prevalence of NAFLD is higher in males [21, 40, 41]. However, the underlying mechanistic basis of sex-specific differences of hepatic steatosis and its clinical consequences are poorly understood.

The SHROB (fa^k^/fa^k^) shows several features of human MetS [6] that include obesity, hyperinsulinemia, hyperlipidemia, and genetically encoded hypertension [23, 24, 26]. Although hormonal and biochemical underpinnings of MetS have been extensively analyzed in SHROB rats, here, we show for the first time that there are gender-specific differences in the development of hepatic steatosis in these animals. Additionally, we show that hyperinsulinemia-induced signaling pathways and a panoply of hepatic genes involved in lipid biosynthesis and metabolism are differentially regulated in male and female SHROB rats.

Consistent with earlier reports, insulin signaling pathways were significantly blunted in SHROB rats, regardless of gender. Nevertheless, SHROB females were significantly more insulin-sensitive. Additionally, we noted gender-specific differences in phosphorylation-dependent activation of p38MAPK, ERK1/2, and SAPK/JNK. The MAPK pathway is characterized by a 3-tier sequential activation of MAPK kinase kinase (MKKK), MAPK kinase (MKK), and MAPK. Extracellular stimuli trigger the MKKK and its downstream cascade of kinases via protein-protein interactions, phosphorylation, and subcellular compartmentalization [34]. The MAPK signaling cascades impinge on transcription factors like ELK-1 [42], PPARγ 2 [43], and SREBPs [44] that regulate gene networks involved in energy homeostasis. Kotzka et al. have shown that the N-termini of SREBP-1a, SREBP-1c, and SREBP-2 are phosphorylated by ERK, JNK, and p38 MAPK [45–48]. The importance of MAPK-mediated phosphorylation of SREBP-1 in pathophysiology of MetS in humans was bolstered by the finding of a rare allele of SREBP-1, defective in serine 117 phosphorylation by ERK and JNK. Patients carrying this mutation elicited severe combined hypolipidemia [49]. Thus, SREBPs converge at the regulatory nexus of insulin resistance, obesity, and inflammation, all of which are subject to regulation by MAPK [50].

Quantification of expression of 45 genes that may contribute to gender-specific differences in hepatic steatosis [37] revealed a rather complex picture of hepatic lipid metabolism in SHROB rats. Thus, genes encoding *Srebf1*, *Acaca*, *Scd*, *Fasn*, and *Acly* were highly induced in the livers of SHROB rats. However, with the exception of *Acaca*, all were similarly induced in the livers of males and females. In contrast, expression of seven genes (*Pparg*, *Cd36*, *Slc2a4*, *Atox1*, *Skp1*, *Angptl3*, *and Pnpla3*) showed a gender-specific bias. The aberrant expression of PPARγ [51] is associated with NAFLD [52–54], and liver-specific loss of PPARγ is known to markedly attenuate the pathogenesis of NAFLD [55, 56]. The PPARγ activates SREBP-1c, a master transcription factor that promotes de novo lipid synthesis [57]. PPARγ2 and C/EBPα cooperate to activate transcription of many adipocyte specific genes [58]. We posit that more enhanced expression of PPARγ in males versus females is mechanistically involved in differential regulation of hepatic steatosis in SHROB rats. PPARα stimulates β-oxidation of fatty acids; PPARα ligands trigger SCD1 activity which is necessary for VLDL secretion [59].

The livers of male SHROB rats expressed lower levels of the *Cd36* gene, consistent with their genetic derivation from Wistar Kyoto (WKR) strain [26, 60]. *CD36* protein belongs to the class B scavenger receptor family [61] and binds to native and oxidized low-density lipoproteins [62] and long-chain fatty acids [63]. The *Cd36* receptor promotes fatty acid uptake into muscle and adipose tissues [64–66]. Deficiency of *Cd36* in SHR rats, shown to be associated with insulin resistance [67, 68], could be corrected by exogenously expressed *Cd36* [69]. Hyperlipidemia and insulin resistance have been reported in *Cd36*-deficient mice [70, 71] and humans [61]. Expression of *Slc2a4* was higher in male SHROB rats putatively causing greater influx of glucose, thus accelerating its flux into the Krebs cycle. Therefore, we posit that enhanced influx of glucose and fatty acids into the livers of male SHROB rats led to more de novo lipid biosynthesis.

All SHROB rats elicited an enhanced expression of *Pnpla3*, but its ~ 500-fold induction in the livers of male rats was remarkable. *Pnpla3* encodes a nonspecific lipid acyl hydrolase that is highly expressed in the liver and adipose tissue [72]. Ninety percent of *Pnpla3* in hepatocytes is associated with lipid droplets [73]; the mechanism by which *Pnapla3* regulates lipid droplet biogenesis remains controversial [74]. Expression of *Pnpla3* is highly induced by carbohydrate feeding and insulin [75]. The promoter of murine *Pnpla3* gene contains consensus binding sites for ChREBP and SREBP-1c [76, 77]. Therefore, it is highly likely that enhanced accumulation of lipid droplets in the livers of SHROB males may be a direct result of elevated expression of ChREBP and SREBP-1c that transcriptionally activated the *Pnlpa3* gene.

The expression of *Atox1* and *Skp1* mRNA was higher in male SHROB rats than female SHROB rats. Induction of *Atox1* and *Skp1* has been associated with high levels of TGs in liver and adipose tissue [37]. *Skp1α* is a component of SCF complex that plays a key role in hepatic lipid accumulation [78]. Hepatic expression of the angiopoietin-like protein 3 (*Angptl3*) mRNA was also higher in SHROB males. *ANGPTL3* is a member of a family of secreted glycoproteins structurally related to angiopoietins that regulates lipid, glucose, and energy metabolism [79–81]. *ANGPTL3* is highly expressed in the liver and acts in concert with LXR [80, 82]. The livers of *db/db* mice expressed high levels of *Angptl3* [83] that could be normalized by leptin treatment [84]. Conversely, targeted deletion of *Angptl3* leads to lower plasma TG and cholesterol [85]. Inactivating mutations in the *ANGPTL3* gene cause familial combined hypolipidemia, a disorder characterized by profound reduction of plasma lipoproteins [86]. Disabling *ANGPTL3* with antisense oligonucleotides or antibodies also lowers plasma TG and LDL cholesterol in humans [87].

## Conclusion

We have corroborated and extended previous observations to conclude that hepatic steatosis in SHROB rats, regardless of their gender, is driven mainly by higher rates of de novo lipogenesis that occur in a setting of conspicuous absence of hyperglycemia [23, 24, 26]. Additionally, our data indicated that female SHROB rats elicited more efficient fatty acid transport (*Cd36*) and esterification (*Pnpla3*), and greater insulin sensitivity (higher phosphorylation of AKT and AMPK), all of which contributed to less severe steatosis. Differential regulation of AMPK in male and female SHROB rats is of great interest since AMPK is known to improve insulin sensitivity by decreasing de novo lipogenesis as well as enhancing both fatty acid oxidation and mitochondrial integrity [88]. A number of recent studies have suggested that a combination of reduced fatty acid oxidation and enhanced rates of de novo lipogenesis might be mechanistically related to differential pathogenesis of NAFLD in humans [21]. Our tentative conclusion must be further moderated by two major caveats. First, the hepatic steatosis in SHROB rats only partially mimics the hepatic consequences of human MetS in light of the dissimilarities in the biology of leptin in rodents and man, where unequivocal involvement of leptin receptor in hepatic steatosis has yet to be demonstrated. Second, an explanation for an apparent lack of markers of overt inflammation and fibrosis, with concomitant induction of SAPK/JNK, in the livers of SHROB rats is puzzling and must await future investigation.

### Perspectives and significance

**Fig. 8:**
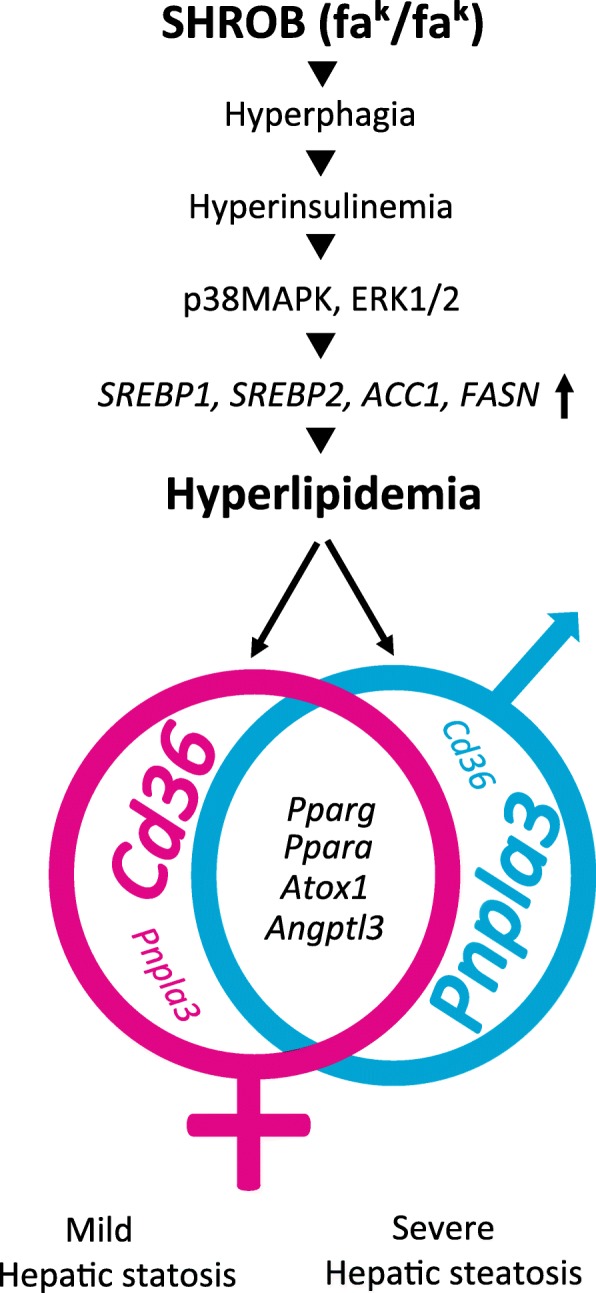
Hypothetical scheme showing major steps involved in sex-specific differences in the pathogenesis of hepatic steatosis in SHROB rats. Both female and male SHROB rats elicit hyperphagia, hyperinsulinemia, and hyperlipidemia. Activation of hyperinsulinemia-induced signaling pathways leads to induction of genes that regulate de novo lipogenesis. Several genes that control lipid metabolism are highly expressed in male and female SHROB rat livers. Expression of *Cd36* and *Pnpla3* genes was differentially regulated in female and male SHROB rats, leading to milder and severe hepatic steatosis, respectively

Sex-specific differences in hepatic steatosis, differential regulation of hyperinsulinemia-associated signaling pathways, and canonical genes involved in lipid metabolism in male and female SHROB rats, outlined here, are highly significant. SHROB rats are hypogonadal, caused by the loss of leptin signaling and a consequential reduction in luteinizing hormone-releasing hormone (LHRH) in the hypothalamus [11, 89]. The prevalence of NAFLD is higher in men with hypogonadism as well in post-menopausal women [90]. However, our hypothetical scenario explaining these data (Fig. 8) provides only a glimpse into the mechanisms involved in hepatic steatosis and its modulation by gender. This mechanistic picture will be considerably sharpened by more comprehensive, genome-wide analyses of hepatic gene expression, using “omics” techniques [91]. Finally, a clear-cut relevance of experimental findings in SHROB rats to gender-specific differences in NAFLD in humans remains murky, particularly in the absence of overt inflammation and fibrosis in this model. A recent report showing that pro-inflammatory responses associated with NAFLD pathogenesis could be exacerbated if mice were housed at their body temperature rather than kept in a conventional, ambient temperature housing [92]. Thus, under thermo-neutral conditions, fatty diet-induced hepatic steatosis was exacerbated in a gender-specific manner in several strains of mice. The notion of temperature-mediated modulation of the pathogenesis of NAFLD in SHROB rats needs to be experimentally tested.

## Additional file

Additional file 1: **Figure S1.** Genotyping of SHROB rats. A: Design of tera-pair primers to amplify leptin receptor region including the T>A mutation by PCR and the resulting sizes of DNA fragments in WT (248-bp and 184-bp), heterozygous (248-bp, 184-bp, and 118-bp) and homozygous (248-bp and 118-bp) are shown. B: An example of targeted sequencing around the mutated base (blue line) from the WT, heterozygous and homozygous genomes are shown. **Table S1.** Primers used in this study. (PDF 1101 kb)

## Abbreviations

Acaca: Acetyl-CoA carboxylase alpha (also known as Acc1)
Acacb: Acetyl-CoA carboxylase beta (also known as Acc2)
ACC1: Acetyl-CoA carboxylase
Acly: ATP citrate lyase
AKT: Serine/threonine kinase (also known as protein kinase B; PKB)
ALT: Alanine aminotransferase
AMPK: AMP-activated protein kinase
Angptl3: Angiopoietin-like protein 3
ANOVA: Analysis of variance
AST: Aminotransferase
Atox1: Antioxidant 1 copper chaperone
BUN: Blood urea nitrogen
Cd36: Cluster of differentiation 36 (also known as fatty acid translocase)
ChREBP: Carbohydrate response-element binding protein
ERK: Extracellular signal-regulated kinase
FASN: Fatty acid synthase
H&E: Hematoxylin and eosin
IRS-1: Insulin receptor substrate 1
JNK: c-Jun NH_2_-terminal kinases
LHRH: Luteinizing hormone-releasing hormone
MAPK: Mitogen-activated protein kinase
MetS: Metabolic syndrome
MKK: MAPK kinase
MKKK: MAPK kinase kinase
MRI: Magnetic resonance imaging
mTOR: Mechanistic target of rapamycin
NAFLD: Non-alcoholic fatty liver disease
NASH: Non-alcoholic steatohepatitis
nSREBP1: Mature sterol regulatory element-binding protein 1
nSREBP2: Mature sterol regulatory element-binding protein 2
p70S6K: Ribosomal protein S6 kinase
Pnpla3: Patatin-like phospholipase domain containing 3
Ppara: Peroxisome proliferator-activated receptor alpha
Pparg: Peroxisome proliferator-activated receptor gamma
pSREBP1: Full-length, precursor sterol regulatory element-binding protein 1
pSREBP2: Full-length, precursor sterol regulatory element-binding protein 2
qPCR: Quantitative polymerase chain reaction
S6: Ribosomal protein S6
Scd: Stearoyl-CoA desaturase (also known as Scd1)
SHR: Spontaneously hypertensive
SHROB: Obese spontaneously hypertensive
Skp1: S-phase kinase-associated protein 1
Slc2a4: Solute carrier family 2 (also known as Glut-4)
Srebf1: Sterol regulatory element-binding transcription factor 1 gene
