# Supplementary material for: Sex-specific differences in hepatic steatosis in obese spontaneously hypertensive (SHROB) rats
Source: Biol Sex Differ. 2018 Sep 10;9:40. doi: 10.1186/s13293-018-0202-x (PMC6131947; doi:10.1186/s13293-018-0202-x)
Supplement: Supplementary file 1 — Figure S1. Genotyping of SHROB rats. A: Design of tera-pair primers to amplify leptin receptor region including the T>A mutation by PCR and the resulting sizes of DNA fragments in WT (248-bp and 184-bp), heterozygous (248-bp, 184-bp, and 118-bp) and homozygous (248-bp and 118-bp) are shown. B: An example of targeted sequencing around the mutated base (blue line) from the WT, heterozygous and homozygous genomes are shown. Table S1. Primers used in this study. (PDF 1101 kb) [file 13293_2018_202_MOESM1_ESM.pdf]

Figure S1 Genotyping of SHROB rats. A: Design of tera-pair primers to amplify leptin receptor region including the T>A mutation by PCR and the resulting sizes of DNA fragments in WT (248-bp and 184-bp), heterozygous (248-bp, 184-bp and 118-bp) and homozygous (248-bp and 118-bp) are shown. B: An example of targeted sequencing around the mutated base (blue line) from the WT, heterozygous and homozygous genomes are shown.

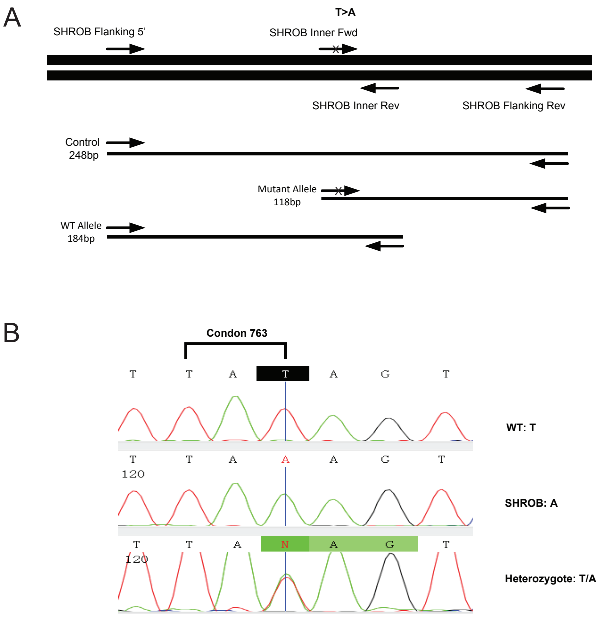

A
T>A
SHROB Flanking 5'
SHROB Inner Fwd
SHROB Inner Rev
SHROB Flanking Rev
Control
248bp
Mutant Allele
118bp
WT Allele
184bp
B
Condon 763
T T A T A G T
WT: T
T T A A A G T
120
SHROB: A
T T A N A G T
120
Heterozygote: T/A

Figure S1

Table S1. *Primers used in this study*

Gene/Primer	Forward (5'-3')	Reverse (5'-3')
SHROB Flanking	GGTTGTTTCTCAATGCAGATAGTAAATT	TAATACTTGTTAACATTCGAAGGGATTC
SHROB Inner	CCTGGACACTGTCACCTAATGATTAAA	TCCATTCAATAACCAGATATAACAGAGTA
Pparg	GCATCAGGCTTCCACTAT	CTTCAATCGGATGGTTCTTC
Ppara	TCTGAATGAGCACTTCTAAG	CCTGTAATTGTCTGAATCCT
Fgf21	GATGGAACTCTCTATGGA	ACATTGTATCCGTCCTTA
Cpt1a	ATATAGATAGATACTGGCTTGT	TTGACTCTGGTTGGAATC
Cpt1b	GAGGCTCTGAGACACATC	CTAAGGATACCATTCTTGATTCG
Ppargc1a	CTTCCTGAACTTGACCTT	CTACACCACTTCAATCCA
Ucp1	ACAATGCTTACAGAGTTA	TACAGTTGATGATGACAT
Adrb3	AATGACTAGACCACAGAA	GTAGTAGCGAAGTTCAAG
Tnf	CCAATCTGTGTCCTTCTAA	TTCTGAGCATCGTAGTTG
Il1b	GGCAACCACTTACCTATT	ACACGGACAACTAGATTC
Il6	TAGTGTGCTATGCCTAAG	TATTGCCAGTTCTTCGTA
Ccl2	TGTAGAAGTGACCAGTATG	AAGTGTTGAACCAGGATT
Adipor1	AGTATGTCCAGGCTTCAAGTAA	ACAGCAGTGATAGCAGGTT
Adipor2	CAGATATAAGGCTCAGAA	TCATAGTAGGAAGAACAA
Cd36	CATAGGACATACTTGGAT	TCTCTTCAGATTCTTCAG
Fabp4	CCGAGATTTCCTTCAAACT	GGTTATGATGCTCTTCACTT
Pdk4	TGCGATCAGTATTATCTAACG	GTGAAGGTGTGAAGGAAC
Slc2a4	TATGTTGCGGATGCTATG	TTAGGAAGGTGAAGATGAAG
Gde1	GAATCCAGGTTGTTAGTTG	ATGCTGTCAGTGATGTAG
Pnpla3	AATATCACCAACCTCAGT	ATCACCTTCACATCAGAT
Atox1	GAGGAGTGGAGTTCAACATTG	GAGAGTTGCCAGCAGGAT
Skp1	AGAAGCGGACAGATGATA	GCAGCCAGAATAAGTTCA
Hnrnpab	GGGTTTATTCTCTTCAAAG	CCTTCTTCATAGCCATAG
Phykpl	GAAGTTCAAGCCTCCAAT	GTCTGTTAGAATGTCATCCA
Invs	GAAGCAGTAATGTAAGTCT	AGGTAGTTCAGTTCTCAA
Abca1	GGATGTATAACGAGCAGTAT	TTAGAGCATTGAGGAGTTC

Lpl	TCTAACTGCCACTTCAAC	CCAACTCTCATACATTCCT
Lipc	GAAGCCAATAGAGCCTAC	TGTCCAGTGTGATAAGTAAG
Mttp	TTAACGGATACAGTGATT	GTCTATTAGCAGAATAAGTC
Arf1	GGAAGACAACAATTCTATAC	GCTGATATTCTTGTATTCAA
Ldlr	ATTCCAAACTCCACTCCAT	CTGCTTCTCATCCTCCAA
Angptl3	CTATGTTGGATGATGTCAA	GATGTGGTTCTTCTTAGC
Pltp	CAGTGCCACAGAGAAGAG	ATGGATTGTAATTGATGGTTGAC
Sort1	TAAGGATACAACAAGAAG	GCCAGAATAGAATAGAAT
Apob	AGCCTGTCAATGTTCTTA	CATCTTGGTTCTTATCGTATT
Apoe	AGGCTAAGGAGTTGTTTC	TTCATCTTCCCAGTTGTG
Apoc3	GGAGCAAGTTCACTGATA	CAAGAGTTGGTGTTGTTAG
Srebf1	GGATTGCACATTTGAAGAC	TGTCTCACCCCCAGCATA
Srebf2	TTGTTGTTGTTGTTGTTATGTT	CGTCAGGTAAGTCCAGAA
Mlxipl	CAACAACAAGATGGAGAAC	GCAGAGTGTCAAATCCTA
Acaca	GAGGTGGATCGGAGATTTCATAG	AGGCTCCAGATGACGATAGA
Acacb	CCTGTCTCAGCCTCCTAGATAA-3	CTGACCAACCTGGTGAAACT
Scd	CCTGCAGAATGGAGGAGATAAG	GCCTTCCTTATCCTTGTAGGTG
Fasn	TACGACTACGGCCCTCATTT	CCATGAAGCTCACCCAGTTATC
Acly	GCTCTGCTACCTGCTGTATTT	CAACATCCTAACGCCCTACAA
Rn18s	AAGACGAACCAGAGCGAAAG	TCGGAACTACGACGGTATCT
